# Methods for Isolation and Purification of Murine Liver Sinusoidal Endothelial Cells: A Systematic Review

**DOI:** 10.1371/journal.pone.0151945

**Published:** 2016-03-18

**Authors:** Jeremy Meyer, Carmen Gonelle-Gispert, Philippe Morel, Léo Bühler

**Affiliations:** 1 Division of Digestive and Transplantation Surgery, University Hospitals of Geneva, Rue Gabrielle-Perret-Gentil 4, 1211, Genève 14, Switzerland; 2 Unit of Surgical Research, University of Geneva, Rue Michel-Servet 1, 1206, Genève, Switzerland; University of Minnesota Medical School, UNITED STATES

## Abstract

To study the biological functions of liver sinusoidal endothelial cells (LSEC) and to identify their interplay with blood or liver cells, techniques allowing for the isolation and purification of LSEC have been developed over the last decades. The objective of the present review is to summarize and to compare the efficiency of existing methods for isolating murine LSEC. Toward this end, the MEDLINE database was searched for all original articles describing LSEC isolation from rat and mouse livers. Out of the 489 publications identified, 23 reported the main steps and outcomes of the procedure and were included in our review. Here, we report and analyse the technical details of the essential steps of the techniques used for LSEC isolation. The correlations between the prevalence of some steps and the efficiency of LSEC isolation were also identified. We found that centrifugal elutriation, selective adherence and, more recently, magnetic-activated cell sorting were used for LSEC purification. Centrifugal elutriation procured high yields of pure LSEC (for rats 30–141.9 million cells for 85–98% purities; for mice 9–9.25 million cells for >95% purities), but the use of this method remained limited due to its high technical requirements. Selective adherence showed inconsistent results in terms of cell yields and purities in rats (5–100 million cells for 73.7–95% purities). In contrast, magnetic-activated cell sorting allowed for the isolation of highly pure LSEC, but overall lower cell yields were reported (for rats 10.7 million cells with 97.6% purity; for mice 0.5–9 million cells with 90–98% purities). Notably, the controversies regarding the accuracy of several phenotypic markers for LSEC should be considered and their use for both magnetic sorting and characterization remain doubtful. It appears that more effort is needed to refine and standardize the procedure for LSEC isolation, with a focus on the identification of specific antigens. Such a procedure is required to identify the molecular mechanisms regulating the function of LSEC and to improve our understanding of their role in complex cellular processes in the liver.

## Introduction

Liver sinusoidal endothelial cells (LSEC) are specific to the liver microcirculation. LSEC line the liver capillaries and transport blood from branches of the portal vein and the hepatic artery into the central vein of liver lobules. They provide a porous barrier between blood components and liver parenchymal cells, i.e., hepatocytes. Moreover their endocytic capacities make them effective scavengers for molecules such as albumin, acetylated low-density lipoproteins (Ac-LDL) and antigens in the bloodstream [1, 2].

At the interface between blood components and parenchymal cells, LSEC are able to interact with various cell types and participate in several physiological and pathological events. For example, LSEC dysregulation is believed to constitute a critical step in liver fibrosis [3] and in non-alcoholic steato-hepatitis progression [4]. Moreover, LSEC have dual roles in liver tissue in the surgical setting, having deleterious effects through involvement in ischemia-reperfusion injury during liver transplantation [5, 6] and beneficial effects through regulating and orchestrating liver regeneration following partial hepatectomy [7]. Notably, we detailed in a recent review the potential role of the interactions between platelets and LSEC in the regenerative process [8, 9].

To study the biological functions of LSEC and to identify the interplay between LSEC and other blood or liver cells, techniques allowing for the isolation and purification of LSEC have been developed over the last decades. Early methods for liver cell dispersion relied on liver tissue mechanical disruption or on differences in cell-specific sensitivity to enzymatic digestion. LSEC enrichment was obtained by isopycnic gradient centrifugation or selective adherence to materials. Currently, published isolation protocols rely on liver tissue enzymatic digestion, discarding parenchymal cells and then further purification of LSEC from the non-parenchymal cell fraction. This final step, in particular, has been affected by the emergence of newer technologies based on the LSEC phenotype [10, 11], such as magnetic-activated cell sorting (MACS) or fluorescent-activated cell sorting (FACS).

In principle, the new approaches were developed to reach higher purities and to shorten the isolation procedure, but limitations have been encountered due to technical requirements and controversies regarding the LSEC phenotype. Moreover, the heterogeneity of isolation protocols, the lack of information regarding the yields and purities of the LSEC population obtained and the absence of standardization of outcome measurement have impeded a comparative methodological evaluation. Furthermore, these issues have revealed a serious problem of possible scientific misinterpretation of experiments due to a low purity [12].

The objective of the present review was to summarize and review the current literature on the existing isolation and purification methods for murine LSEC. We carefully explored the different techniques and compared the major outcomes of LSEC isolation, cell yields and purities, and discussed these methods in regard to the recent findings in the field, notably regarding the LSEC-specific phenotype.

## Materials and Methods

The present methodology is in accordance with the Preferred Reporting Items for Systematic Reviews and Meta-Analyses (PRISMA) statement [13] (S1 Table).

### Inclusion Process

An extensive literature search was conducted using the MEDLINE database for original articles related to murine LSEC isolation that were published up until the 18^th^ of July 2015. The search build is reported in Table 1. The abstracts, or if not concluding, full texts, were screened. All articles in English reporting original data about LSEC isolation from healthy rat or mouse livers were considered for inclusion. The methods and results sections of these publications were then read to confirm definitive inclusion according to pre-defined criteria. The articles that reported the following data were included: 1) the digestion method (including the digestion step and the enzyme used), 2) the method for discarding parenchymal cells, 3) the method for LSEC purification, 4) the cell yield per liver, and 5) the purity of the final cell preparation.

**Table 1:** MEDLINE search build.

(liver sinusoidal endothelial cell [Title/Abstract] **OR** liver sinusoidal endothelial cells [Title/Abstract] **OR** liver endothelial cell [Title/Abstract] **OR** liver endothelial cells [Title/Abstract] **OR** liver sinusoidal cell [Title/Abstract] **OR** liver sinusoidal cells [Title/Abstract] **OR** sinusoidal endothelial cell [Title/Abstract] **OR** sinusoidal endothelial cells [Title/Abstract])	**AND**	(isolation [Title/Abstract] **OR** isolated [Title/Abstract] **OR** purification [Title/Abstract] **OR** primary [Title/Abstract])	**AND**	(rat [Title/Abstract] **OR** rats [Title/Abstract] **OR** mouse [Title/Abstract] **OR** mice [Title/Abstract] **OR** murine [Title/Abstract])

### Variables of Interest

The following data were extracted from the included publications: 1) the date of publication, 2) the type of study, 3) the species and strains of the animals, 4) the animal and liver statuses during the digestion step, 5) the access to the liver for perfusion, anticoagulation and liver washing prior to digestion (referred as *liver preparation*), 6) the enzyme and type of digestion (referred as *liver digestion*), 7) the filtration of the digested liver tissue, 8) the method for removal of parenchymal cells, 9) the method for LSEC purification, 10) the LSEC yield per liver, 11) the method used to estimate the purity of the final cell preparation at the end of the procedure, 12) the viability and 13) purity of the final cell preparation, and 14) the proportion of contaminating resident macrophages. If not explicitly provided in the text, the cell yield per liver was calculated according to the data provided by the authors for an estimated liver weight of 10 grams for rats [14] and 1.5 grams for mice [15]. If several strains or groups were described, only the animals receiving no treatment prior to the isolation procedure or referred to as controls were considered for the analysis.

## Results

### Inclusion Process

**Fig 1:**
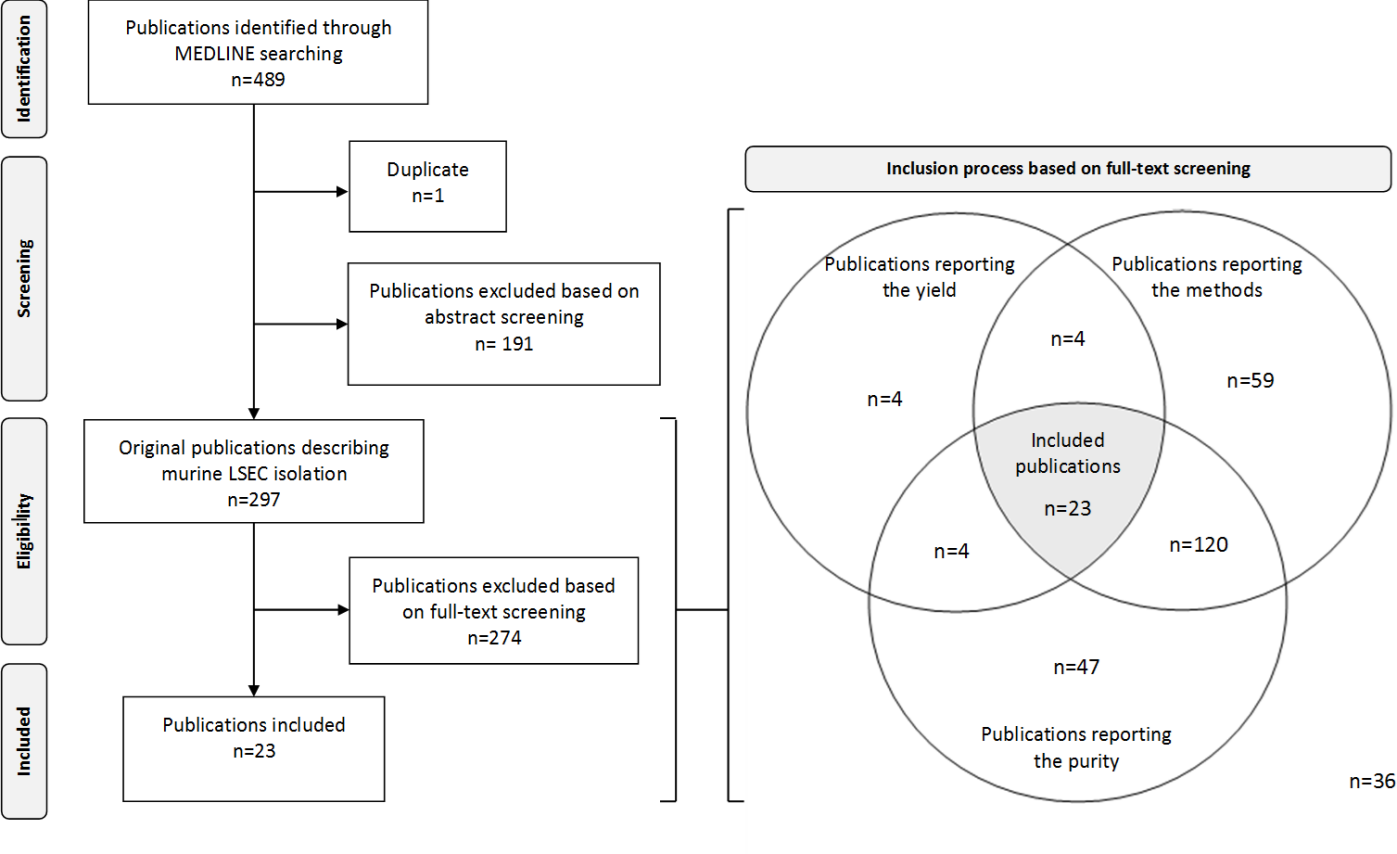
Flow diagram of the inclusion process.

The inclusion process is summarized in Fig 1. A total of 489 publications were identified using a keyword search of the MEDLINE database. Of the articles that were identified, 192 were not considered further for inclusion. Among them, 135 were original publications that did not describe murine LSEC isolation, 33 described LSEC isolation from altered or injured livers, 12 were written in foreign languages, 11 were review articles and 1 publication was reported twice by the keyword search. Among the 297 original publications describing murine LSEC isolation, 274 were excluded for not reporting the main outcomes of the procedure, as detailed in Fig 1. Ultimately, 23 publications were included for further analysis.

### Preparation of the Liver for Enzymatic Digestion

The first step of all modern LSEC isolation procedures relies on liver cell dispersion using enzymatic digestion of liver tissue [16]. The liver can be either incubated in an enzyme-containing solution or, since the introduction of liver physiological perfusion by Berry and Friend [17], perfused with an enzyme-containing solution. This technique, which consists of infusing the liver through the portal vein with a washing solution followed by a solution containing the enzyme, is referred as the two-step digestion technique. The washing solution can be supplemented or not with an anticoagulant drug to prevent the formation of thrombi that could impair the subsequent enzyme perfusion.

#### Animal and liver statuses

The procedure was carried out in anesthetized animals in all studies reporting LSEC isolation from rats [12, 18–21]. One publication reported a two-step perfusion procedure, with *in situ* Pronase perfusion, followed by *ex situ* Collagenase perfusion [22]. Conversely, 85.71% (6 out of 7 reporting the animal status) isolation procedures from mice were performed using previously sacrificed animals [23–28]. Nevertheless, in these studies, 83.33% (5/6) of the livers were perfused *in situ*. Only one publication reported liver excision and *ex situ* enzymatic perfusion [28].

#### Liver vascular access

Most livers were perfused through the portal vein in both species (11/12 reporting liver access). Either 22- [27], 24- [25] or 30-gauge [24] catheters were used in mice, while 18-gauge catheters were used in rats [19, 20]. In both species, the inferior *vena cava* was described to be sectioned to allow for efflux of the perfusion solutions [19, 20, 23, 27]. Other authors reported accessing the liver through the portal vein, but did not provide technical details of this step [22, 26, 29]. Notably, one study described a retrograde approach in rats, associating the cannulation of the infra-hepatic portion of the inferior *vena cava* with a 16-gauge catheter, the ligation of the supra-hepatic portion of the inferior *vena cava* and the section of the portal vein [21].

#### Liver preparation for digestion

100-200UI of heparin were administered intravenously in 4 out of 9 studies reporting data regarding the liver preparation in rats [12, 19, 20, 30]. Livers were then perfused with either Gey’s balanced salt solution for 5 minutes [22], calcium-free Gey’s balanced salt solution for 10 minutes [12], calcium-free Krebs-Ringer solution supplemented with EGTA for 10 minutes [31], 200ml of calcium-free Gey’s balanced salt solution [19, 20], 30ml of calcium-free Hank’s balanced salt solution supplemented with EGTA [18], 75ml of L-15 salts [30] or with an undefined saline solution [21].

In contrast, 5 of 8 studies did not describe a pre-perfusion step of the liver [23–26, 28] and none reported preliminary anticoagulation in mice. Of those preparing the liver for enzymatic perfusion, Sun et *al*. used calcium-magnesium-free Hank’s balanced salt solution [29], whereas Liu et *al*. reported using calcium-free Gey’s balanced salt solution for 10 minutes [27] and Do et *al*. calcium-free Gey’s balanced salt solution for 15–20 minutes [32].

### Liver Cell Dispersion

To obtain efficient liver cell dispersion, the surface of contact between the liver tissue and the enzymatic solution has to be maximized. Most authors provide access to the liver tissue by performing a physiological perfusion through the portal vein. In contrast, incubation in an enzyme-containing solution was sometimes reported.

In rats, liver tissue digestion was mostly performed using liver perfusion with Collagenase at concentrations ranging from 0.03% to 0.05% [31, 33–38]. Of note, some authors reported using an enzyme recirculation system to avoid the loss of dispersed cells [12, 22, 30]. The perfusion step could be followed by incubation in the same solution that was associated [19, 20] or not [18, 21, 22] with DNase for as much as 30 minutes [18, 22]. Another study described the use of Pronase for liver digestion [39].

Procedures for liver digestion were slightly different in mice, as most authors relied on an incubation phase for liver cell dispersion. For example, Deleve et *al*. [23] and Topp et *al*. [24] perfused the liver for a short time with Collagenase diluted in a calcium-free medium before incubating the excised organ in a calcium-supplemented solution of Collagenase for 30 minutes. Katz et *al*. [25] and Schrage et *al*. [26] perfused small volumes (2ml) of enzyme solution (Collagenase±DNase) into the portal vein before incubating the excised liver into the same solution for 20 minutes. Slightly differently, Liu et *al*. [27] performed both liver perfusion and incubation with the enzyme solution for 10 minutes each. Of note, another group prolonged the liver digestion phase to 1.5 hours by incubation in a 0.1% Collagenase type II solution without performing any preliminary perfusion step [28].

In both species, undigested liver tissue was removed using a unique filtration with 50–297μm meshes [19, 20, 24, 25, 28, 32, 37], or by successive passages through meshes of different sizes [27, 38]. Although this tissue was usually discarded, it could be reused for further processing and digestion [30].

### Isolation of Non-Parenchymal Cells from Digested Liver Tissue

Following liver cell dispersion, digested liver tissue is mainly composed of parenchymal cells. Removal of these undesired cells can be achieved by centrifuging the digestion product at low speed to pellet the hepatocytes, by performing gradient centrifugation to separate parenchymal cells from non-parenchymal cells based on their respective densities, or by a combination of both techniques.

Non-parenchymal cells were separated from parenchymal cells solely using differential centrifugation of the digested liver tissue in 3 out of 23 (13.04%) studies [18, 31, 36]. Furthermore, differential centrifugation that was associated with a one- or two-step gradient was used in 13 out of 23 (56.52%) studies [19–21, 24, 25, 27, 29, 32, 33, 35–38] and with two successive gradients in 1 (4.35%) study [34]. Differential centrifugation was usually performed 1–3 times at 50xg for 2 minutes, but was also performed successively at 10 and 16xg [31], three times at 70xg [36], or once at 100xg for 5 to 10 minutes [19, 20, 29]. One-step gradients were composed of Metrizamide [25, 35], Nycodenz [24, 34], Percoll [32] or Optiprep [25]. Two-step gradients were mainly made of 25/50% Percoll [19–21, 29, 33, 34, 36–38]; however in a more recent study, these gradients were composed of 8.2/17.6% Optiprep [27]. Three publications reported non-parenchymal cell isolation and parenchymal cell discard using a one-step isopycnic centrifugation in 17.5% Metrizamide [23], 26% Nycodenz [26] or 17% Iodixanol [12].

In rats, another method for non-parenchymal cell isolation described in the 1980s consisted of liver perfusion and digestion by Pronase to destroy parenchymal cells, in association with centrifugation in a one-step gradient of Metrizamide [22, 39] or a four-step gradient of Stractan [30].

Notably, in one study, LSEC purification using CD31+ MACS was directly performed without preparing a non-parenchymal cell population [28].

### Liver Sinusoidal Endothelial Cell Purification

LSEC purification refers to the extraction of LSEC from the non-parenchymal cell fraction. This step is thought to be critical, as the contamination by non-parenchymal cells other than LSEC, such as macrophages or stellate cells, could impact the results of primary cells-based experiments. LSEC purification can be achieved by segregating LSEC from undesired cells using their differences in density (centrifugal elutriation), their functional properties–such as their ability to quickly adhere to materials (selective adherence)–or, more recently, their phenotypic characteristics (MACS or FACS).

#### For rats

LSEC were purified from the cell fraction devoid of parenchymal cells by centrifugal elutriation in 37.50% of the publications (6/16) [12, 18, 22, 31, 35, 39], by selective adherence in 43.75% (7/16) [19–21, 30, 33, 34, 36] and by SE-1-based MACS in 6.25% (1/16) [36]. No purification step following gradient centrifugation was reported in 2 publications [37, 38]. Short-term (approximately 20 minutes) selective adherence was performed to remove contaminating macrophages from the preparation [36], while long-term (2 hours or more) selective adherence was chosen to remove non-adherent cells, such as contaminating lymphocytes or neutrophils [33]. Four out of 7 authors (57.14%) practicing selective adherence combined both short- and long-term selective adherences to obtain better purities of the final cell preparation [19–21, 34]. But also, a long-term selective adherence of 48 hours followed by trypsinization was used to separate macrophages from LSEC because macrophages are supposed to be less sensitive to trypsin [30].

#### For mice

No included publication reported short-term selective adherence as a method for LSEC purification in mice, whereas centrifugal elutriation from preparations devoid of parenchymal cells was used in 25% (2/8) of the studies [23, 24]. MACS represented the preponderant technique used to separate LSEC from other non-parenchymal cells in mice. Sorting was performed according to published phenotypic characteristics of LSEC to select CD11b- CD54+ cells [32], CD45- cells in a two-step procedure [25], CD146+ cells [26, 27] or CD31+ cells [28]. In some studies, MACS was followed by long-term selective adherence to further increase the purity of the final cell preparation by removing contaminating non-adherent cells that were retained in the sorting columns [26, 32]. Rather unusual, one publication did not report a further purification step after gradient centrifugation [29].

### Methods for Purity Estimation of the Final Cell Preparation

Methods for purity estimation of the final cell preparation obtained by liver digestion and processing relied on either functional or phenotypic characteristics of LSEC. For instance, the phagocytic capacities of LSEC have been widely described and used to characterize LSEC in most studies. The uptake of amine-conjugated ovalbumin [34, 37] and the uptake of Ac-LDL alone [21, 26] were both reported to identify LSEC. To evaluate purity and to distinguish between LSEC and contaminating macrophages, counter-stains such as endogenous peroxidase [12, 23, 24, 29, 31] and non-specific esterase [33] were used, and the macrophage ability to ingest labelled-Staphylococcus aureus [30] or latex beads [31] was reported. Regarding the morphology of LSEC, the identification of fenestrations at the cell surface using transmission electron microscopy was performed to determine the purity in 3 studies [19, 20, 22]. Immunofluorescence or immunohistochemistry for RECA-1 and von Willebrand factor (vWF) [38], or CD32b (targeted by the SE-1 antibody) were reported in rats [18, 36], while flow cytometry for CD45- [25], CD31+ [27] or CD31+ and CD45+ [28] cells were performed in mice. One study described LSEC as cells negative for peroxidase staining [35]. Another study performed a modified Wright-Giemsa staining [32]. Knook et *al*. relied on undefined cytochemical and ultrastructural characteristics [39].

### Outcomes of the Isolation Procedures

The main outcomes of any isolation procedure are documented by the number (referred to as cell yield), purity and viability of cells obtained. The methods to estimate the purity are mainly based on either functional or phenotypic characteristics of the desired cells, as previously discussed.

#### For rats

Isolation procedures based on the removal of parenchymal cells by Pronase digestion, followed by Metrizamide gradient centrifugation and purification by centrifugal elutriation yielded the highest quantities of LSEC in rats, which ranged from 100 to 160 million cells per liver [22, 39]. Purity was assessed using “cytochemical and ultrastructural characteristics” [39] but also more precisely using transmission electron microscopy [22]. Other publications reporting isolation using centrifugal elutriation, preceded or not by differential centrifugation and gradient centrifugation, allowed for the acquisition of 30–60 million cells per liver [18, 31, 35] and more than 120 million cells if the centrifugal elutriation was performed twice [12]. Purity was assessed using the Ac-LDL uptake assay in association with peroxidase staining [12, 31] or immunofluorescence using SE-1 [18], but also by only negative staining for peroxidase [35].

When purification was performed using short-term selective adherence, associated or not with long-term selective adherence, the yields were lower and ranged between 23.9 and 40 million cells per liver [19–21, 34, 36]. Purity was estimated to be >95% when assessed using the Ac-LDL uptake assay alone [21] or by immunohistochemistry using SE-1 [36]. Purity was >80% when estimated using the amine-conjugated ovalbumin uptake assay [34], and approximately 70% when determined by transmission electron microscopy [19, 20]. Long-term selective adherence alone allowed for higher yields (60–100 million cells per liver) with an estimated >95% purity according to the Ac-LDL uptake assay in association with non-specific esterase staining, although the removal of macrophages after gradient centrifugation was not described [33]. Long-term selective adherence associated with selective trypsinization at 48 hours after plating procured approximately 35 million cells per liver with an estimated purity of 90% [30].

When cells were retrieved from Percoll gradient centrifugation without any further purification step, the yields ranged from 32 to 75 million cells per liver with >90% purities, according to the amine-conjugated ovalbumin uptake assay [37] and immunofluorescence for RECA-1 and vWF [38]. Purification using SE-1-based MACS allowed for the acquisition of approximately 11 million LSEC with a purity of 97.6% [36].

When reported, viability was above 90% for all procedures [12, 19, 20, 30, 31, 35, 36, 39], except for one that did not include any LSEC purification step [38].

#### For mice

Similarly, the highest cell yields were reported in mice when LSEC purification was performed using centrifugal elutriation. Approximately 9 million cells per liver were retrieved with >95% purity, as estimated by the Ac-LDL uptake assay coupled to peroxidase staining [23, 24]. Surprisingly, >98% purity for 7.5–10.5 million cells was obtained when no purification step was performed following gradient centrifugation [29]. The cell yields were lower when using MACS-based purification methods. For instance, CD11b- CD54+ MACS allowed for the acquisition of 0.5 million cells with an estimated purity of 90% [32], while CD45- and CD146+ techniques isolated 1–5 million LSEC per liver with similar purities [25–27]. In contrast, CD31+ MACS yielded 7.5–9 million cells [28].

The methods for purity estimation were heterogeneous and mainly relied on LSEC phenotype. The functional Ac-LDL uptake assay was only used once, and was not used in combination with peroxidase staining [26], while flow cytometry for CD31 and/or CD45 was used in most studies [25, 27, 28]. Surprisingly, some authors assessed purity by estimating the proportion of CD45- cells [25] while others used CD45+ cells [28]. When reported, the viability was above 90% [24, 27, 29].

## Discussion

Historically, techniques for liver cell dispersion relied on non-enzymatic methods, such as liver tissue mechanical disruption, in combination or not with calcium, magnesium or potassium chelators. However, the cells thus obtained were damaged and did not maintain their functional properties [40, 41]. The introduction of both enzymatic liver dispersion by Howard et *al*. [16] and liver physiological perfusion by Berry and Friend [17], allowed for the acquisition of preserved cells in high yields. The technique, which was initially described in rats, consists of infusing the liver through the portal vein with a calcium-free solution followed by a calcium-supplemented solution containing Collagenase and Hyaluronidase, and has been widely used and adapted by many groups [41].

Collagenases are endopeptidases derived from *Clostridium histolyticum* that digest native collagen in the triple helix region [42], which allow for cell dispersion. Collagenases are classified based on their enzymatic activities, stabilities and amino acid compositions, and constitute the most commonly used enzymes for liver digestion at concentrations that usually range from 0.01 to 0.08% [37, 40, 41], as illustrated in Tables 2 and 3. Although Collagenase was initially associated with Hyaluronidase [41, 43], the use of this association has not been described in recent publications. Furthermore, to prevent the formation of cell aggregates, DNase was added to the enzymatic solution during perfusion and/or incubation [19, 20] and also during consecutive steps [30] in some studies.

**Table 2:** Baseline characteristics of included publications regarding liver sinusoidal endothelial cell isolation from rats

Authors	Year	Strain	Animal status	Liver status	Liver access	Liver preparation	Liver digestion	Filtration	Removal of parenchymal cells	Gradient	LSEC purification	Yield (nx10^6^)	Estimation of purity	Purity (%)	Macrophages (%)	Viability (%)
Smedsrod et *al*. [37]	1985	Sprague-Dawley	-	-	-	-	P: Collagenase	50μm	Differential centrifugation	Percoll	None	75†	IF: amine-conjugated ovalbumin uptake	90–95%	-	-
Thiele et *al*. [38]	1999	Wistar	-	*In situ*	-	None	P: 0.05% Collagenase IV	205μm 105μm	Differential centrifugation	25/50% Percoll	None	32±5.4	IF: RECA-1+ vWF	96.4%	<5%	>85%
Knook et *al*. [39]	1981	BN/BiRij	-	*-*	-	-	P: Pronase; I: Pronase	-	(Pronase)	Metrizamide	Centrifugal elutriation	141.9±12.1†	Cytochemical and ultrastructural characteristics	>90%	-	93%
De Leeuw et *al*. [22]	1982	BN/BiRij	-	*In situ + ex situ*	PV	P: GBSS	P: 0.2% Pronase E; P: 0.05% Collagenase I + 0.05% Pronase E; I: 0.05% Collagenase I + 0.2% Pronase E	Yes	(Pronase)	Metrizamide	Centrifugal elutriation	100–160	TEM	90–95%	1–5%	-
Smit et *al*. [35]	1987	Wistar	-	*-*	-	-	P: Collagenase	-	Differential centrifugation	Metrizamide	Centrifugal elutriation	30–50	IF: peroxidase (negative staining)	93%	7%	>90%
Moriga et *al*. [18]	2000	Wistar	A	*In situ*	PV	P: Calcium-free HBSS	P: 0.05% Collagenase; I: 0.05% Collagenase	None	Differential centrifugation	None	Centrifugal elutriation	30–50	IF: SE-1 antibody	>95%	-	-
Krause et *al*. [31]	2000	Wistar	-	*In situ*	-	P: Calcium-free Krebs-Ringer solution	P: 0.03% Collagenase	None	Differential centrifugation	None	Centrifugal elutriation	40–60	IF: Ac-LDL uptake + peroxidase/latex beads phagocytosis	85–90%	5%	>90%
Deleve et *al*. [12]	2006	Sprague-Dawley	A	*In situ*	-	200 UI Heparin; P: Calcium-free GBSS	P: 0.05% Collagenase 1a	Yes	None	17% Iodixanol	Centrifugal elutriation (2x)	>120‡	IF: Ac-LDL uptake + peroxidase	>98%	-	>95%
Friedman et *al*. [30]	1987	Sprague-Dawley	A	*In situ*	-	100 UI Heparin ; P: L-15 salts	P: 0.2% Pronase; P: 0.015% Collagenase; I: 0.02% Pronase + 10μg/ml DNase	Yes	(Pronase)	6/8/12/20% Stractan	SA: 24 hours; Trypsinization 48 hours after initial plating	35.0±2.9	IF: Ac-LDL uptake + *Staphylococcus aureus* uptake	90.4±3.6%	-	96.8±3.1%
Heldin et *al*. [34]	1991	Sprague-Dawley	-	*-*	-	-	P: Collagenase	None	Differential centrifugation	13% Nycodenz; 30/50% Percoll	SA: 10 minutes ; SA: 20 hours	40	IF: amine-conjugated ovalbumin	>80%	-	-
Yannariello-Brown et *al*. [33]	1992	Sprague-Dawley	-	*-*	-	-	P: Collagenase	-	Differential centrifugation	25/50% Percoll	SA: 2 hours	60–100	IF: Ac-LDL + non-specific esterase	>95%	-	-
Braet et *al*. [19]	1994	Wistar	A	*In situ*	PV	150 UI Heparin; P: Calcium-free GBSS	P: Collagenase; I: 0.05% Collagenase A + 0.001% DNase	100μm	Differential centrifugation	25/50% Percoll	SA: 20 minutes ; SA: 2 hours	23.9±3.7	TEM	73.7±5.8%	12–16%	95–98%
Braet et *al*.[20]	1995	Wistar	A	*In situ*	PV	150 UI Heparin; P: Calcium-free GBSS	P: 0.05% Collagenase A + 0.001% DNase; I: 0.05% Collagenase A + 0.001% DNase	100μm	Differential centrifugation	25/50% Percoll	SA: 20 minutes ; SA: 2 hours	25	TEM	75%	12%	95–98%
Pollok et *al*. [21]	1998	Lewis	A	*In situ*	IVC	P: Saline solution	P: 0.05% Collagenase D ; I: 0.05% Collagenase D	None	Differential centrifugation	25/50% Percoll	SA: 20 minutes ; SA: 2 hours	5–30	IF: Ac-LDL uptake	>95%	-	-
Tokairin et *al*. [36]	2002	Fischer 344	-	-	-	-	P: Collagenase; I: Collagenase	-	Differential centrifugation	25/50% Percoll	SA: for macrophages	24.3±0.5	IHC: SE-1 antibody	92.0±0.8%	2.3±0.5%	93.7±1.4%
Tokairin et *al*. [36]	2002	Fischer 344	-	-	-	-	P: Collagenase; I: Collagenase	Yes	Differential centrifugation	None	MACS: SE-1-based	10.7±0.5	IHC: SE-1 antibody	97.6±0.9%	0.9±0.1%	94.0±1.3%

A = Anesthetized, GBSS = Gey's Balanced Salt Solution, HBSS = Hank's Balanced Salt Solution, I = Incubation, IF = Immunofluorescence, IHC = Immunohistochemistry, LSEC = Liver Sinusoidal Endothelial Cell, MACS = Magnetic-Activated Cell Sorting, P = Perfusion, PV = Portal Vein, SA = selective adherence, TEM = Transmission Electron Microscopy, S = Sacrificed, vWF = von Willebrand Factor

† Calculated with an estimated liver weight of 10 grams

‡ Cell yield per liver was calculated according to the data provided by the authors

**Table 3:** Baseline characteristics of included publications regarding liver sinusoidal endothelial cell isolation from mice.

Authors	Year	Strain	Animal status	Liver status	Liver access	Liver preparation	Liver digestion	Filtration	Removal of parenchymal cells	Gradient	LSEC purification	Yield (nx10^6^)	Estimation of purity	Purity (%)	Macrophages (%)	Viability (%)
Sun et *al*. [29]	2007	C57BL/6	-	-	PV	P: Calcium-free HBSS	P: 0.5% Collagenase A; I: 0.5% Collagenase A	None	Differential centrifugation	25/50% Percoll	None	7.5–10.5†	IF: Ac-LDL uptake + peroxidase	>98%	-	>95%
Deleve [23]	1994	C_3_H/HE	S	*In situ*	PV	None	P: 0.05% Collagenase Ia; I: 0.05% Collagenase Ia	Yes	None	17.5% Metrizamide	Centrifugal elutriation (2x)	9	IF: Ac-LDL uptake + peroxidase	>95%	-	-
Topp et *al*. [24]	2004	SvEv	S	*In situ*	PV	None	P: Collagenase Ia; I: Collagenase Ia	297μm	Differential centrifugation	29% Nicodenz	Centrifugal elutriation	9.25±2.47	IF: Ac-LDL uptake + peroxidase	>95%	-	>95%
Do et *al*. [32]	1999	Balb/c	A	*In situ*	PV	P: GBSS	P: Liver digest medium; I: 0.02% Collagenase type 4 + 0.0005% DNase	75μm	Differential centrifugation	Percoll	MACS: CD11b-; MACS: CD54+; SA: 2 hours	0.5	Morphological characteristics	90%	2%	-
Katz et *al*.[25]	2004	C57BL/6; BALB/c; OT-II Rag-2-/-	S	*In situ*	PV	None	P: 1% Collagenase ; I: 1% Collagenase D	100μm	Differential centrifugation	30% Metrizamide or 40% Optiprep	MACS: CD45- (2x)	2.5±0.62	FC: CD45-	99%	-	-
Schrage et *al*. [26]	2008	C57BL/6; CXCR3-/-	S	*In situ*	PV	None	P: 2mg/ml Collagenase IV + 0.2mg/ml DNase I; I: 2mg/ml Collagenase IV + 0.2mg/ml DNase I	Yes	None	26% Nycodenz	MACS: CD146+; SA: overnight	1–5	IF: Ac-LDL uptake	95–98%	-	-
Liu et *al*. [27]	2011	C57BL/6	S	*In situ*	PV	P: Calcium-free GBSS	P: 0.16mg/ml Collagenase IV; I: 0.16mg/ml Collagenase IV + 10μg/ml DNase I	150μm; 100μm; 70μm	Differential centrifugation	8.2/17.6% Optiprep	MACS: CD146+	2.6±0.4	FC: CD31+	91.7±2.1%	4.3%	94.3±2.1%
Chou et *al*. [28]	2015	C57BL/6	S	*Ex situ*	None	None	I: 0.1% Collagenase II	100μm	None	None	MACS: CD31+	7.5–9†	FC: CD31+ CD45+	94.4±2.3% (CD31); 82.5±4.7% (CD45)	-	-

A = Anesthetized, FC = Flow Cytometry, GBSS = Gey's Balanced Salt Solution, HBSS = Hank's Balanced Salt Solution, I = Incubation, IF = Immunofluorescence, LSEC = Liver Sinusoidal Endothelial Cell, MACS = Magnetic-Activated Cell Sorting, P = Perfusion, PV = Portal Vein, SA = selective adherence, S = Sacrificed

† Calculated with an estimated liver weight of 1.5 grams

Liver preparation through perfusion with a saline solution in combination or not with administration of an anticoagulant therapy with heparin (systemic or in situ) was performed in the majority of studies for rats and in 3 out of 8 studies for mice. The perfusion step allowed for the blood to be flushed out of the liver to prevent the formation of thrombi, which is one of the main causes of inadequate liver perfusion and digestion [40].

Moreover, Seglen et *al*. reported that calcium removal before enzyme perfusion, using chelating agents or efficient washout with a calcium-free buffer, facilitated the subsequent separation of cells by Collagenase infusion [44]. Although most research groups carried out this infusion step [12, 18–20, 27, 29, 31], some publications did not report pre-perfusion of the liver [23–26, 28, 38] or reported performing pre-perfusion using buffers that were not depleted in calcium [21, 22, 30, 32]. Similarly, although Collagenase is a calcium-dependent enzyme, some authors reported the use of digestion solutions that were depleted in calcium [23, 24]. In conclusion, it appears that the two-step Collagenase perfusion technique is the most used procedure to obtain digested liver tissue. Whether derivative methods such as incubation of the excised liver in an enzymatic solution or the use of recirculation systems, as described in some studies [12, 22, 30], represent an improvement of the procedure, remains to be formerly evaluated.

The initial cell suspension obtained after liver cell dispersion contains, in addition to intact non-parenchymal cells, high numbers of parenchymal and damaged cells. Methods for purifying non-parenchymal cells from hepatocytes allow for a drastic reduction in the processed volume for subsequent procedures and optimize purification. Non-parenchymal cells could be obtained by selective destruction of parenchymal cells using proteolytic enzymes or, as mostly applied, by differential and/or isopycnic centrifugation.

Pronase, a protease derived from *Streptomyces griseus* [40], selectively destroys parenchymal cells and allows for the isolation of LSEC in high yields, as reported in Table 2 [22, 30, 39]. In the literature, livers were either perfused with Pronase [22, 30, 39], with a mixture of Pronase and Collagenase [45] and/or incubated with Pronase [30, 41]. Non-parenchymal cells obtained by Pronase dispersion were, however, described as having altered attachment efficiency [46, 47], survival [47] and endocytic capacities [40], and currently this technique is not recommended.

Because non-parenchymal cells are much smaller than hepatocytes, and damaged parenchymal cells are much lighter, hepatocytes can also be separated from non-parenchymal cells by differential pelleting. Parenchymal cells are sedimented at a low centrifugation speed by repeating the centrifugation for 3 or 4 times, and then non-parenchymal cells are recovered from the supernatant [41]. Blomhoff et *al*. compared the recovery of non-parenchymal cells from liver digest products isolated from rats by Collagenase digestion followed by differential centrifugation, to the recovery after incubation with Pronase or enterotoxin. The latter method gave high yields of viable cells, in contrast to differential centrifugation. However, cells retrieved by differential centrifugation showed a higher proportion of LSEC compared to macrophages [48]. Considering what we have discussed, differential centrifugation is the most commonly used technique for non-parenchymal cell isolation among the publications included in our review [18–21, 24, 25, 27, 29, 31–38] and in the literature, while methods using the selective destruction of parenchymal cells have not gained in popularity.

Similarly, non-parenchymal cells can be further purified according to density using isopycnic gradient centrifugation. Early methods reported using one-step gradients composed of Ficoll [43], Metrizamide [22, 23, 35, 39, 49], Nycodenz [24, 26, 50], Iodixanol [12] or Percoll. Due to their lower weight, non-parenchymal cells could be separated from remaining parenchymal cells and damaged cells following Pronase treatment and from blood cells, which all sedimented to the bottom of the tube, while non-parenchymal cells remained at the top of the gradient. The limit of isopycnic sedimentation is that most non-parenchymal cells have densities that overlap. In contrast, discontinuous gradients allow cells to be separated over a wide range of densities, causing cells to accumulate at interfaces between these densities. These gradients not only allow for the purification of non-parenchymal cells but also allow for fractionation between their subpopulations [27]. Discontinuous gradients reported in the literature are 6/8/12/20% Stractan [30], 6/8/12/15% Larex [51, 52], 8.2/15.6% Accudenz [53, 54], 6/8/12/16% Larcoll [55, 56], 11/15% Metrizamide [57], 8.2/16% Optiprep [27, 58] and the commonly used 25/50% Percoll [19–21, 29, 33, 38]. Some authors also reported performing 2 consecutive gradients, such as one-step gradient of 13% Nycodenz followed by 30/50% Percoll [34]. The efficiency of the density separation by the different gradients appeared similar, which indicated that the use of a two-step gradient, such as Optiprep, might be adequate for the isolation of non-parenchymal cells. However, because macrophages and LSEC have densities that overlap, attempts to separate them by means of density-gradient centrifugation only have remained unsuccessful. For this reason, most recent techniques include additional purification steps.

Centrifugal elutriation, which is a technique that was introduced in the 1970s [59, 60], was first used for LSEC purification in rats, and the results for cell yield, purity and viability were excellent, as summarized in Tables 2 and 3. Centrifugal elutriation is usually performed after parenchymal cell removal using a combination of both enzymatic destruction by Pronase and isopycnic centrifugation in Metrizamide [22, 39], by differential centrifugation and Metrizamide [35] or by isopycnic centrifugation alone [12]. The highest cell yields were obtained with the Pronase technique [22, 39]. Among the techniques without Pronase, yields of 30–60 million cells per liver were obtained in rats by removing parenchymal cells through differential centrifugation [18, 31, 35], while yields of >120 million cells were obtained when centrifugal elutriation was performed twice after isopycnic centrifugation in 17% Iodixanol [12].

Even though elutriation resulted in high yields of LSEC, this technique is a time-consuming procedure that requires expensive equipment and technical support. Moreover, average purities were sometimes reported [61]. The first affordable/accessible methods developed for LSEC isolation relied on the difference in the speed of adhesion of macrophages and LSEC to culture dishes. These methods of selective adhesion were first described in rats [19–21, 34, 36] before being modified for mice [62]. They methods are mostly preceded by gradient centrifugation, to remove contaminating cells that could impair the purification step [19–21, 30, 33, 34, 36]. Both short-term (until 20 minutes) [36] and long-term (more than 2 hours) [33] selective adherences have been described to remove macrophages and blood cells, respectively. In several studies, both adherence steps were combined [19–21, 34]. Moreover, trypsinization at 48 hours after initial plating (long-term adherence) was described to allow for the selective recovery of LSEC because macrophages are less sensitive to trypsin [30]. These adherence techniques seem to be less efficient than centrifugal elutriation in terms of both cell yield and purity, as illustrated in Table 2. For instance, the purities of the LSEC populations obtained, as analysed by transmission electron microscopy, were of 73.7±5.8% [19] and 75% [20]. These preparations contained high proportions of contaminating macrophages (12–16%), white blood cells and other unidentified cells [19, 20]. These findings demonstrate that selective adherence is an unsatisfactory approach for the purification of LSEC from the non-parenchymal cell fraction.

During the last decades, many molecules were reported to be expressed on LSEC and have become essential for discriminating LSEC from endothelial cells of the periportal and centrilobular areas. Immunofluorescence and immunohistochemistry studies from rats that were included in the present review revealed that RECA-1 [38], vWF [38] and CD32b [18, 36] are expressed on LSEC. In mice, LSEC were shown to be CD31+ [27], CD45- [25] or CD31+ CD45+ cells [28] using flow cytometry. More phenotypic markers were described in the literature to assess the purity of the final cell preparations. These markers are CD44 [63], CD105 [64], ET-1 [65, 66], vimentin [67] and the scavenger hyaluronan receptor stabilin-2 [68, 69] in rats; CD40 [70], CD80 [71], CD86 [71], HLA class II [25, 70], fms-like tyrosine kinase [72], foetal liver kinase [72] and vWF [72] in mice; and CD14 [65, 66, 70, 73], CD31 [64, 70], CD34 [72, 74], CD54 [25, 63, 72, 74] and CD106 [70, 72, 74] in both species.

Moreover, surface antigens allowed the development of cell-sorting technologies for LSEC purification, such as MACS or FACS. To our knowledge, the first publication reporting the immuno-sorting of LSEC was published in 1999 [32]. Since then, depending on the published phenotypes, methods for MACS-based LSEC purification have been reported using CD32b+ [36, 75], CD19+ [66], CD31+ [65], stabilin-2+ [68] MACS in rats, and CD146+ [26, 27, 58, 76], CD31+ [28], CD105+ [71, 77], CD45- [25], CD11b- CD54+ [32], CD45- CD146+ [78–80] MACS in mice. Similarly, FACS for CD31+ cells was reported in rats [81], and FACS for CD146+ [82] or CD45- CD146+ cells [80] were reported in mice.

These sorting techniques are almost always preceded by steps for removing parenchymal cells and several types of non-parenchymal cells [25–27, 32, 36] because the presence of high numbers of unlabelled cells might compromise the specific retention of the magnetic-labelled cells in the columns, resulting in a cell population of low purity.

Tokairin et *al*. compared the outcome of MACS purification based on a positive selection of cells recognized by the SE-1 antibody to the outcome of the purification based on isopycnic centrifugation in a 25/50% Percoll gradient in combination with short-term selective adherence. Non-parenchymal cells obtained by a two-step Collagenase perfusion and differential centrifugation were separated into two groups for further purification in parallel procedures. While the technique based on gradient centrifugation and selective adherence allowed for the acquisition of 24.3±0.5 million cells, the LSEC purity only reached 92.0±0.8 according to immunohistochemistry using SE-1, which was mainly due to contamination by macrophages (2.3±0.5%) and stellate cells (0.7±0.2%). In contrast, SE-1-based MACS led to the purification of 10.7±0.5 million cells with a purity of 97.6±0.9% [36]. Moreover, Deleve et *al*. compared the outcomes of gradient centrifugation followed by CD31+ MACS with centrifugal elutriation for LSEC purification. They found that CD31+ MACS yielded 0.17±0.01 million cells, and centrifugal elutriation 100.67±5.07 million cells, from 20% and 80% of the non-parenchymal cell fractions, respectively [12]. To conclude, while MACS allows for a shorter purification process and provides a relatively high cell purity, the cell yields remain unfortunately lower than those obtained by other techniques.

Although it is commonly thought to alter viability of sensitive cells, FACS has also been used for LSEC purification. Bartnek et *al*. isolated non-parenchymal cells from mouse liver using Collagenase perfusion and incubation followed by differential centrifugation and 25/50% Percoll gradient centrifugation. They further purified LSEC into CD45- and CD146+ cells using either MACS or FACS. While the purity was better using FACS (97.45±0.70% *versus* 80.03±8.06%), the recovery rate, i.e., the proportion of cells recovered from the non-parenchymal cell fraction, was inferior to MACS (12.13±12.51% *versus* 30.37±21.20%). The viability was similar for both purification techniques (88.13±4.14% *versus* 80.03±8.06%) [80].

Of note, the expression of several of the reported phenotype markers for LSEC remains controversial. This controversy might be due to the use of LSEC isolated from diseased or aged livers that are subjected to capillarization or from livers subjected to specific types of treatments. LSEC phenotypes might also be different in experiments performed *in vivo* and *in vitro* [10, 11].

These variations could lead to inappropriate immuno-sorting and characterization of LSEC. For instance, CD146, which is widely used for LSEC isolation by MACS, was not restricted to LSEC, but it is also expressed on the endothelium of various mouse tissues and in a subset of NK cells [26]. Therefore, some investigators introduced a double sorting for CD45- CD146+ cells to prevent the retention of blood-borne cells [78–80]. Similarly, CD31-based immuno-sorting and purity assessment were recently found to be inappropriate, as CD31 was shown to be highly expressed on the central and portal tract endothelia, while its expression on LSEC was low [10, 83, 84]. Importantly, cultured CD31+ cells that were sorted by MACS did not exhibit the sieve plates, which is a specific morphological characteristic of LSEC [12, 85].

Moreover, immuno-sorting using a surface antigen introduces the potential risk of the selecting only a subpopulation of LSEC. This might be the case for the expression of CD45 on rat LSEC. We found that both positive [28] and negative [25] selections of cells using CD45 labelling were proposed to characterize and purify LSEC, respectively. This is not surprising, as LSEC have been described to present CD45+ high, CD45+ low and CD45-negative subpopulations in rats [86]. These observations raise doubts regarding the identity of the isolated cells, and highlight the need for the identification of a surface antigen that is specifically expressed by LSEC.

In rats, the SE-1 antibody targets the antigen CD32b, which is a low-affinity Fcγ receptor that is involved in the phagocytosis of immune complexes and specifically expressed on rat LSEC [83, 87]. Evidence of this specificity was shown using immunofluorescence and immunohistochemistry, and confirmed using the functional Ac-LDL uptake assay and scanning electron microscopy [18, 36]. Moreover, LSEC purification using CD32b+ MACS has been reported [36, 75].

Furthermore, the expression of CD32b was associated with the hyaluronan receptor stabilin-2 in rats [69], which is a receptor expressed on the discontinuous endothelium in human and rat livers, spleens and lymph nodes [88–90]. This receptor was also used to characterize LSEC using flow cytometry and immunocytochemistry in rats [68, 69], and for LSEC isolation by MACS [68]. Both these receptors allow for the unambiguous distinction of endothelial cells, macrophages and LSEC and can be therefore considered as reliable marker for LSEC in rats.

In mice, stabilin-2 was shown to be expressed on the endothelial sinuses of the liver [91]. A further characterization of this antigen and its specificity for LSEC in mice might help to further improve the techniques for isolating LSEC from mice.

In addition to their phenotypic characteristics, LSEC have historically been identified based on their unique endocytic capacities. Assays for ovalbumin [34, 37] or Ac-LDL [12, 21, 30, 31, 33] uptakes have been widely described in the publications we have reviewed as methods to determine LSEC purity. However, Ac-LDL was reported to be partially uptaken by macrophages *in vivo* [92] and *in vitro* [2, 93, 94], and more than 10% of cells were shown to be positive for the macrophage marker F4/80 out of a population that was 98% positive for Ac-LDL uptake [93], demonstrating that the Ac-LDL endocytic capacity is not specific to LSEC. Therefore, assessing purity with only this method could have been confounded by macrophage contamination in the cell preparations. To prevent this issue, the Ac-LDL uptake assay was often used in combination with counter-stains for endogenous peroxidase [12, 23, 24, 29, 31], non-specific esterase [33] or with an assay for the uptake of *Staphylococcus aureus* [30], which allowed for the distinction of LSEC from macrophages. Moreover, Ac-LDL was reported to be uptaken from all microvascular endothelial cells, irrespective of their sinusoidal origin [95]. To determine purity, other publications relied on “cytochemical and ultrastructural characteristics” [39], morphological characteristics under phase contrast light microscopy [32] or negative staining for macrophages [35], with the risk of encountering difficulties in distinguishing LSEC from contaminating cells with similar morphologies.

Considering the potential shortcomings related to LSEC identification, Deleve et *al*. recently proposed new methods for LSEC isolation to validate their results using electron microscopy, to show that the majority of cells obtained have fenestrae organized in sieve plates [12].

For the present review, we performed an extensive screening of all publications reporting murine LSEC isolation allowing us to carry out, for the first time to our knowledge, a comparative description of existing techniques in terms of outcomes and a careful analysis of reliability of the procedure that was used. We addressed the existing controversies regarding the specific phenotype of LSEC, which revealed the need for a more standardized technique for LSEC immuno-sorting and characterization. The main shortcoming of this review is the impossibility of performing a direct comparison between the existing techniques, notably because of the heterogeneity of the methods encountered at all of the steps of the isolation and characterization procedures. However, the meticulous lecture of the protocols and the information provided by the literature in the field allowed us to identify trends and to provide recommendations for future practice.

In conclusion, many different procedures have been described for the isolation of LSEC in mice and rats. The two-step liver physiological perfusion allows for efficient liver cell dispersion, as it maximizes the exposition of liver tissue to the action of the digesting enzyme. The use of Pronase should be discontinued as it is deleterious to the survival and functions of isolated cells, in favour of Collagenase, in combination or not with DNase. The choice of the method to discard parenchymal cells and isolate non-parenchymal cells should be left at the discretion of the teams working in the field, as no method was shown to be superior to the other. Nevertheless, this step remains critical as it allows to reduce the volume of the processed cells for the remainder of the procedure. Regarding LSEC purification from the non-parenchymal cell fraction, and despite the heterogeneity in outcome measurement, it turned out that, while centrifugal elutriation and selective adherence have been used for decades for LSEC isolation, MACS constitutes the most promising and accessible technique for LSEC isolation, notwithstanding the low cell yields sometimes reported. The controversies regarding some of the phenotype markers used for LSEC should raise some caution about their application, particularly when using markers that are not specific to LSEC. While a standardized and efficient method for MACS-based LSEC isolation is still lacking in mice, reliable methods have emerged for the isolation of LSEC in rats, which notably use SE-1-based [36, 75] or stabilin-2+ [68] MACS with good results. Similarly, reliable markers for LSEC that can distinguish LSEC from contaminating macrophages and/or endothelial cells are still needed, and should be *sine qua non* for the isolation and characterization for LSEC from mice. We suggest to validate their specificity using the functional Ac-LDL uptake assay in combination with a counterstain for macrophages, or using electron microscopy imaging to demonstrate the presence of fenestrae [12].

In general, more effort is needed to describe the outcomes of LSEC isolation procedures, in terms of cell yield and purity, to highlight the efficiency of the methods used. Notably, out of 297 publications reporting murine LSEC isolation, purities and cell yields were not reported in 67 (22.56%) and 226 (76.09%) studies, respectively, while 36 (12.12%) provided no information. Reporting the outcomes will allow for a meaningful interpretation of the results that are obtained using isolated LSEC. Moreover, further studies are needed that compare the methods used for LSEC isolation, similar to those of Deleve et *al*. [12], Tokairin et *al*. [36] and Bartneck et *al*. [80], and will be of great interest for studying the role and functions of LSEC.

## Supporting Information

S1 Table PRISMA checklist. (DOC)

## Abbreviations

Ac-LDL: Acetylated low-density lipoprotein
FACS: Fluorescence-activated cell sorting
LSEC: Liver sinusoidal endothelial cell
MACS: Magnetic-activated cell sorting
vWF: von Willebrand factor
